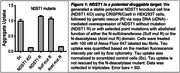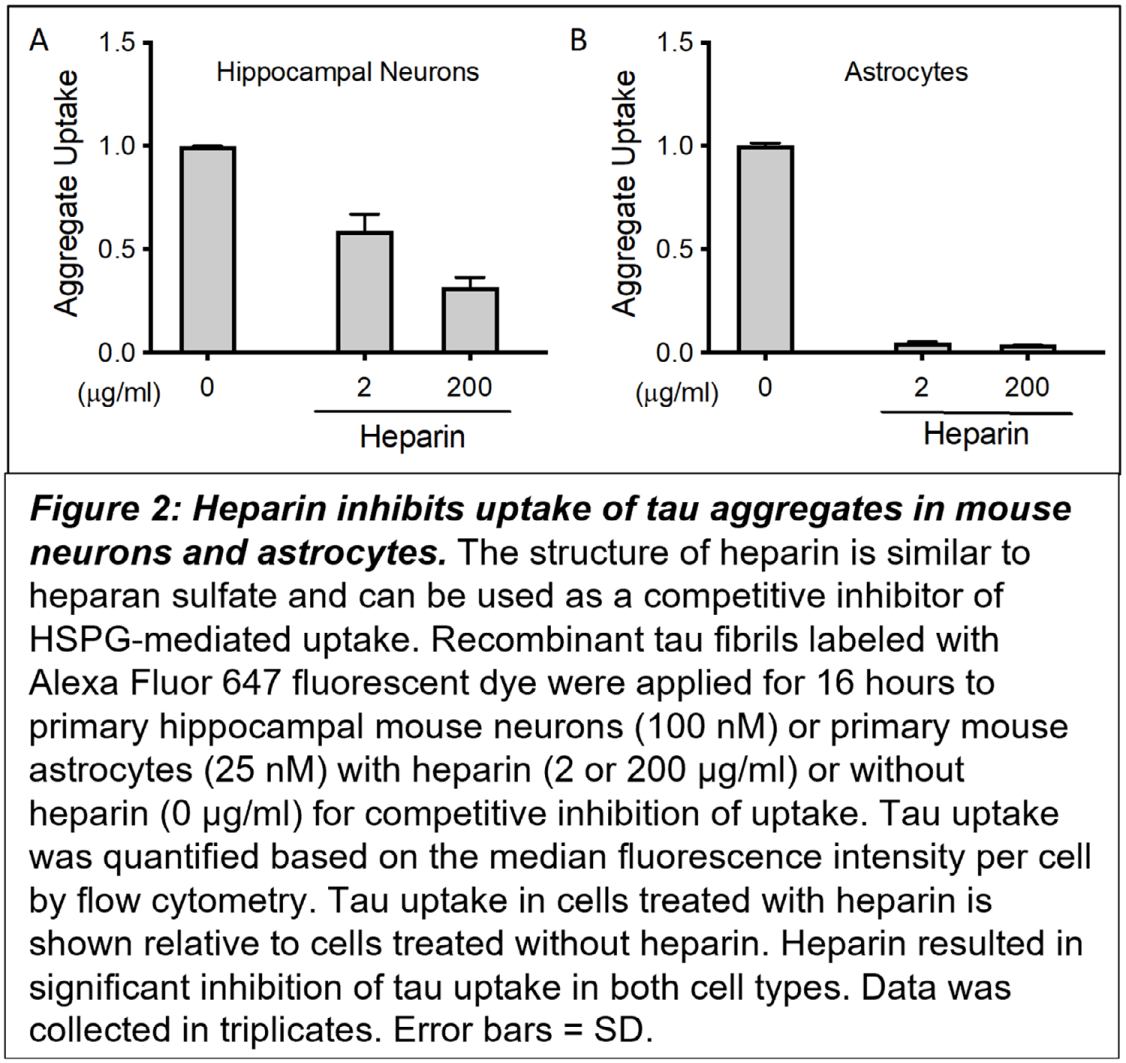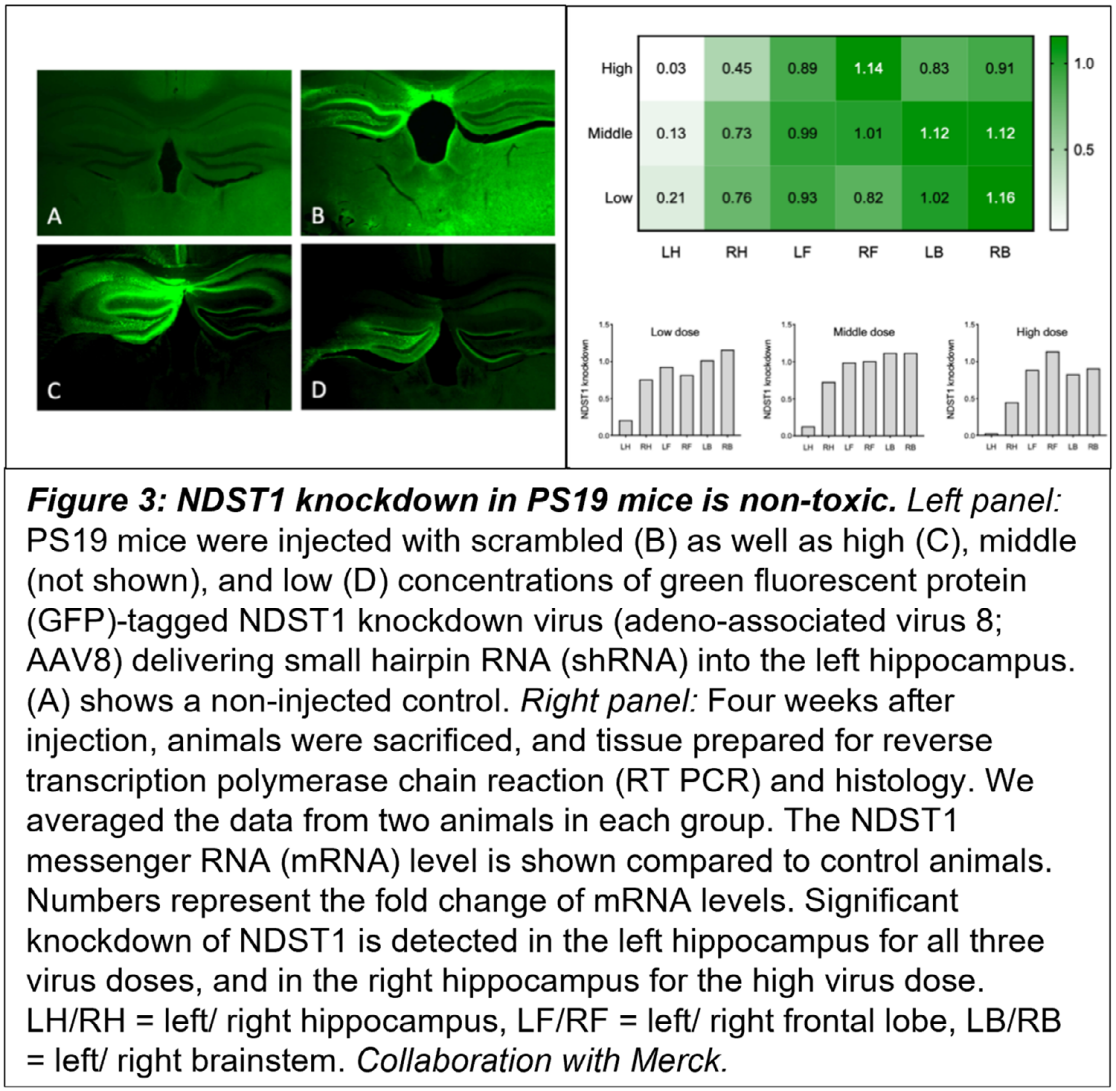# The role of heparan sulfate proteoglycans for tau pathology in vivo

**DOI:** 10.1002/alz.086006

**Published:** 2025-01-03

**Authors:** Barbara E Stopschinski, Sandi‐Jo Estill‐Terpack, Sushobhna Batra, Shubhangi Pandey, Jaime Vaquer‐Alicea III, Marc I Diamond

**Affiliations:** ^1^ University of Texas Southwestern Medical Center, Dallas, TX USA

## Abstract

**Background:**

The prion model of tau propagation in Alzheimer’s Disease predicts that tau seeds are released from cells and taken up by neighboring cells, resulting in spreading of the disease. Our previous work revealed that tau aggregates bind to heparan sulfate proteoglycans (HSPGs) on the cell surface, followed by cellular uptake via macropinocytosis. HSPGs are glycoproteins, consisting of a protein core and decorated with linear glycosaminoglycan (GAG) chains called heparan sulfate (HS) with highly variable sulfation patterns. We have previously used a combination of biochemical, pharmacological and genetic tools to demonstrate that N‐sulfation (mediated by the N‐sulfotransferase NDST1) of the HS chain is critical for tau uptake in cell models. This project is a stepwise approach to validate HSPGs and specifically NDST1 as a therapeutic target to block tau spread in vivo.

**Method:**

We induced NDST1 knockout using CRISPR/Cas9 followed by rescue experiments and tau uptake assays in HEK293T cells. Tau uptake assays with heparin were conducted in murine hippocampal neurons and astrocytes. We employed stereotactic adeno‐associated virus 8 (AAV8)‐mediated intracerebral delivery of small hairpin RNA (shRNA) to target NDST1 in PS19 mice, a tauopathy mouse model (collaboration with Merck).

**Result:**

Our data demonstrates that reduction of the N‐deacetylase activity with selected point mutations within NDST1 can decrease tau uptake substantially (Figure 1). Our experiments in primary murine neurons and astrocytes suggest that HSPGs are crucial for tau uptake in these cell types (Figure 2). NDST1 knockdown with shRNA in PS19 mice achieved >75% reduction of messenger RNA (mRNA) levels and was non‐toxic (Figure 3).

**Conclusion:**

Our findings suggest that NDST1 is a potentially druggable target, and that the HSPG pathway plays a role for tau uptake in multiple cell types. We worked with GemPharmatech for the generation of a floxed NDST1 mouse line. We plan to breed the floxed NDST1 mice to PS19 mice and selected neuronal, astrocytic and microglial Cre driver lines. We anticipate that knockout of NDST1 in vivo will reduce tau spread in mouse brain. If successful, our study will inspire multiple lines of follow up work such as the design of enzymatic inhibitors and genetic silencing tools.